# Higher AJCC stage at primary tumor diagnosis may predict shorter survival in metastatic uveal melanoma

**DOI:** 10.1038/s41598-025-03961-1

**Published:** 2025-06-04

**Authors:** Serdar Yavuzyigitoglu, Shiva Sabazade, Viktor Gill, Erwin Brosens, Emine Kiliç, Gustav Stålhammar

**Affiliations:** 1 Department of Ophthalmology, Erasmus MC Cancer Institute, Erasmus MC, Rotterdam, The Netherlands; 2 Division of Eye and Vision, Department of Clinical Neuroscience, Karolinska Institutet, Eugeniavägen 12, 17164 Stockholm, Sweden; 3 Ocular Oncology Service, St. Erik Eye Hospital, Stockholm, Sweden; 4 Department of Pathology, Västmanland Hospital Västerås, Västerås, Sweden; 5 Clinical Genetics, Erasmus MC Cancer Institute, Erasmus MC, Rotterdam, The Netherlands; 6 St. Erik Ophthalmic Pathology Laboratory, St. Erik Eye Hospital, Stockholm, Sweden

**Keywords:** Uveal melanoma, Choroidal melanoma, Stage, Survival, Metastasis, Prognosis, Eye cancer, Melanoma, Cancer, Oncology

## Abstract

In this international collaboration, we examined whether survival in metastatic uveal melanoma is associated with primary tumor size at initial diagnosis. A total of 128 and 205 patients with American Joint Committee on Cancer (AJCC) stage IV disease were included from the Netherlands and Sweden, respectively. Across both cohorts, patients whose tumors had a largest basal diameter ≥ 16 mm or a higher AJCC stage presented with a greater number of hepatic metastases and showed shorter Kaplan-Meier overall survival from the time of metastatic detection. However, this association did not persist after adjustment for the number of hepatic metastases in multivariate Cox regression and a Markov multi-state model. These findings suggest that primary tumor size is associated with survival in metastatic disease and the burden of metastatic lesions at detection, indicating a potential survival benefit of primary tumor treatment. Nevertheless, caution is warranted when interpreting these results, as they differ from some previous reports.

**Supplementary Information:**

The online version contains supplementary material available at 10.1038/s41598-025-03961-1.

## Introduction

Uveal melanoma is the most common primary intraocular malignancy in adults, characterized by its high propensity for developing fatal metastases^1^ The long-term incidence of metastatic death approaches 50%, with fewer improvements in prognosis compared to cutaneous melanoma^2,3^.

Primary tumor size and anatomic extent are critical factors in the American Joint Committee on Cancer (AJCC) staging system, which strongly predicts the time to metastatic disease^4,5^ In 2014, Damato and colleagues demonstrated that among those who succumb to their disease, the median survival time from primary tumor treatment to death was 4.6 years for patients in stage I, compared to only 2.7 years for patients in stage III^6^ Thus, a shorter metastasis-free interval is associated with poorer overall survival outcomes^7–10^.

However, the shorter time to metastasis and death in patients with more advanced stages might simply reflect lead-time bias, with larger tumors having had more time to grow and metastasize^11^ In this scenario, the treatment of large and small tumors might occur at different points on the same timeline, potentially resulting in similar dates of death despite different initial tumor sizes.

To address this issue, we propose evaluating the survival time from the detection of metastases to death. If this interval is shorter for patients whose primary tumors were larger at the time of treatment, it would suggest that the growth rate of metastases is associated with the size of the primary tumor. This would imply a survival benefit of treating the primary tumor at an earlier stage. In turn, this would clarify the potential benefit of treating uveal melanoma as soon as possible after diagnosis.

However, previous studies have not identified an association between primary tumor diameter at initial diagnosis and an increased risk or hazard ratio for death among patients who develop metastases^7,8,12,13^ The largest study to date, which did not include time-to-event survival analyses, included 330 patients with metastatic choroidal and ciliary body melanomas^12^ While this study confirmed a relationship between a shorter metastasis-free interval and reduced overall survival, it found no differences in AJCC stage distribution among patients with varying overall survival times.

In the present study, we analyze data from 333 metastatic patients treated at two European centers. Our aim is to determine whether primary tumor size influences survival after the onset of metastatic disease.

## Results

### Descriptive statistics

Of the 333 included patients with metastatic disease, 176 (53%) were female. The mean age at the time of diagnosis of the primary tumor was 61 years (SD 12 years). The mean largest basal diameter (LBD) was 13.2 mm (SD 3.5 mm), the mean tumor thickness was 6.7 mm (SD 3.0 mm), and the mean tumor volume was 609 mm³ (SD 493 mm³). Further details on the included patients and tumors are provided in Table 1.

**Table 1:** Characteristics of included patients and tumors.

		All patients		Rotterdam cohort		Stockholm cohort	
Variable	Category	*n*	(%)	*n*	(%)	*n*	(%)
Sex	Female	176	53	69	54	107	52
	Male	157	47	59	46	98	48
AJCC T-category*							
	T1a	38	11	14	11	24	12
	T2a	112	34	31	24	81	40
	T2b	12	4	12	9		
	T2c	1	< 1	1	< 1		
	T2d	4	1	3	2	1	< 1
	T3a	93	28	34	27	59	29
	T3b	17	5	16	12	1	0
	T3c	5	2	3	2	2	< 1
	T3d	5	2	5	4		
	T4a	16	5	5	4	11	5
	T4b	8	2	3	2	5	2
	T4d	1	< 1	1	1		
	N/a	21	6			21	10
AJCC Stage*							
	I	35	11	14	11	21	10
	IIA	108	32	31	24	77	38
	IIB	105	32	46	36	59	29
	IIIA	42	13	28	22	14	7
	IIIB	13	4	8	6	5	2
	IIIC	1	< 1	1	< 1		
	IV	11	3			11	5
	N/a	18	5			18	9
Kaplan-Meier metastasis-free survival, median years (95% CI)		2.0 (1.9–2.2)		2.2 (1.9–2.7)		2.0 (1.6–2.2)	
Kaplan-Meier overall survival after detection of metastases, median years (95% CI)		1.0 (0.8–1.2)		0.6 (0.4–0.8)		1.3 (1.1–1.5)	

*At the time of primary tumor diagnosis. AJCC, American Joint Committee on Cancer. CI, confidence interval.

All patients underwent baseline radiological examinations. In the Netherlands, abdominal ultrasound and chest x-ray were the first-choice imaging modalities. When these were inconclusive, additional imaging with computed tomography (CT), or magnetic resonance imaging (MRI) was performed. In Sweden, CT of the thorax and abdomen was the first-choice imaging modality. Of the 333 patients, 11 (3%) had metastases at initial presentation. Among these 11 patients, 4 (36%) had large primary tumors (LBD ≥ 16 mm). Two hundred and twenty-two patients (67%) underwent regular (biannual) liver ultrasonography for at least five years after primary tumor treatment. Among those with large primary tumors (LBD ≥ 16 mm), 48 of 76 (63%) received regular surveillance, compared with 174 of 245 (71%) for smaller tumors (LBD < 16 mm; chi‐square *P* = 0.20). Patients in surveillance did not present with a greater number of hepatic metastases at the initial diagnosis of metastatic disease (chi‐square test for trend *P* = 0.26). However, those with large primary tumors (LBD ≥ 16 mm) or more advanced AJCC stage had significantly more hepatic metastases upon radiological detection (chi‐square test for trend *P* = 0.01 and *P* < 0.001, respectively, Tables 2 and 3).

**Table 2:** Number of hepatic metastases by primary tumor diameter.

	1 metastasis	2–5 metastases	6–10 metastases	> 10 metastases
Primary tumor LBD				
< 16 mm (*n*)	16	31	10	33
≥ 16 mm (*n*)	4	4	7	20

LBD, largest basal diameter. The metastases counts indicate the number of hepatic lesions detected by radiological examination at the initial diagnosis of metastatic disease. Chi-square test for trend, *P* = 0.01.

**Table 3:** Number of hepatic metastases by AJCC stage.

	1 metastasis	2–5 metastases	6–10 metastases	> 10 metastases
Stage				
I (*n*)	3	4	1	5
II (*n*)	14	24	9	28
III (*n*)	3	7	7	20

AJCC, American Joint Committee on Cancer. LBD, largest basal diameter. The metastases counts indicate the number of hepatic lesions detected by radiological examination at the initial diagnosis of metastatic disease. Chi-square test for trend, *P* < 0.001.

Visual inspection showed that the log-minus-log survival curves were parallel and did not cross, suggesting that the proportional hazards assumption was adequately met for the AJCC stage covariate (Supplementary Figure S1).

### Prognostic implication of primary tumor size for survival in metastatic disease

Among the 333 included patients, AJCC stage at the time of primary tumor diagnosis was significantly associated with survival after the detection of metastases. The median Kaplan-Meier survival estimates after the first radiological detection of metastases were as follows: Stage I, 1.43 years (95% CI, 0.80–2.15); Stage IIA, 1.08 years (95% CI, 0.88–1.48); Stage IIB, 0.99 years (95% CI, 0.72–1.34); Stage IIIA, 0.40 years (95% CI, 0.30–0.75); Stage IIIB, 0.86 years (95% CI, 0.54–not determinable); Stage IIIC, 0.08 years (95% CI, not determinable); and Stage IV, 0.41 years (95% CI, 0.27–not determinable). The 1year survival rates by AJCC stage were as follows: Stage I, 58.9% (95% CI, 44.5–78.0); Stage IIA, 52.7% (95% CI, 44.0–63.2); Stage IIB, 49.5% (95% CI, 40.8–60.0); Stage IIIA, 29.3% (95% CI, 18.2–47.1); Stage IIIB, 46.2% (95% CI, 25.7–83.0); and Stage IV, 27.3% (95% CI, 10.4–71.6). Both patients initially diagnosed with Stage IIIC disease died within a year of metastasis detection.

**Fig. 1:**
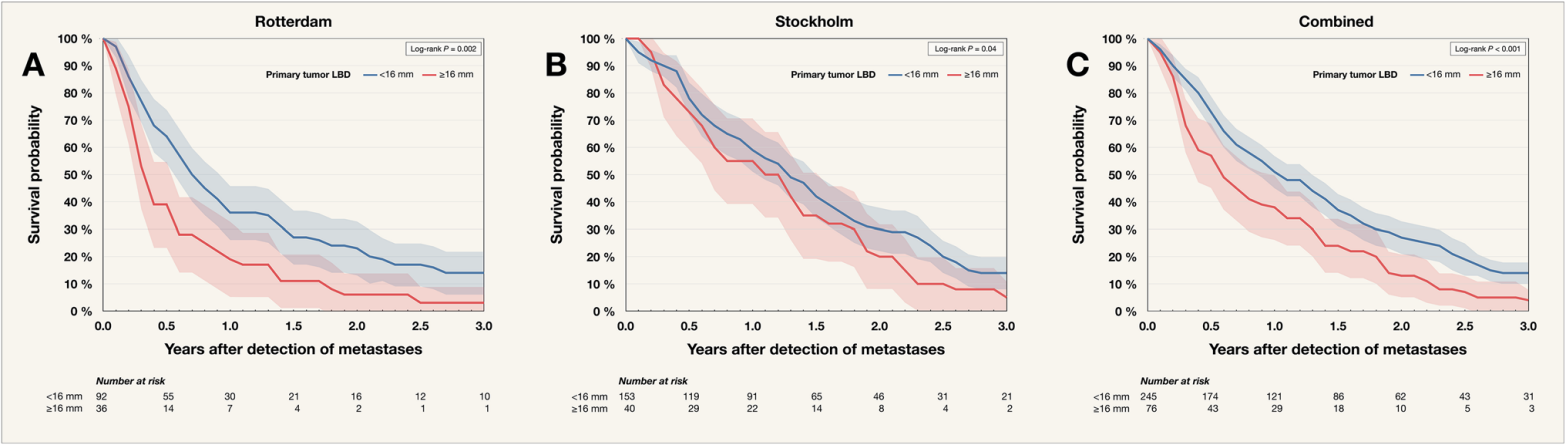
Kaplan–Meier survival curves for 321 patients with metastatic uveal melanoma, stratified by the largest basal diameter (LBD) of the primary tumor. Twelve patients without LBD data were excluded. (**A**) Rotterdam (the Netherlands) cohort, (**B**) Stockholm (Sweden) cohort, and (**C**) combined cohorts. In both cohorts, patients with LBD < 16 mm at the time of treatment had significantly longer survival after metastasis detection than those with LBD ≥ 16 mm. *P* values were corrected using the Holm–Bonferroni method. AJCC, American Joint Committee on Cancer.

**Fig. 2:**
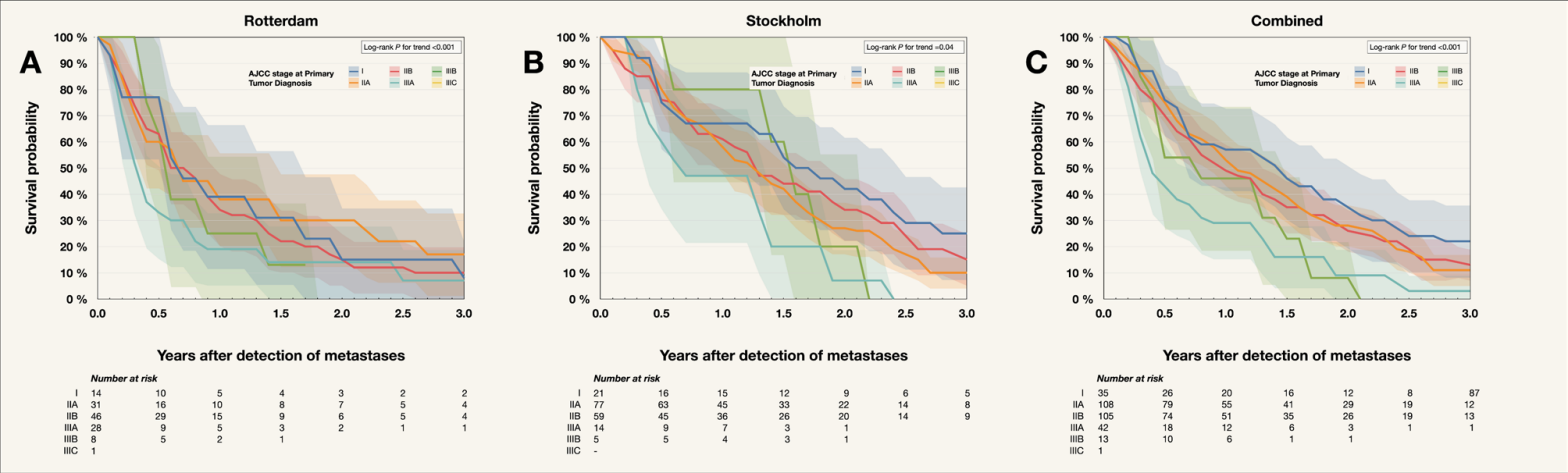
Kaplan–Meier survival curves for 222 patients with metastatic uveal melanoma, categorized by AJCC stage at the time of primary tumor diagnosis. Eleven patients already in stage IV at diagnosis were excluded. (**A**) Rotterdam cohort, (**B**) Stockholm cohort, and (**C**) combined cohorts. *P* values were corrected using the Holm–Bonferroni method. AJCC, American Joint Committee on Cancer.

In both cohorts, patients with primary tumors having an LBD < 16 mm demonstrated longer survival after metastasis detection (log-rank *P* ≤ 0.04, Fig. 1). Similarly, when stratified by AJCC stage, a significant trend was observed in both cohorts (log-rank *P* ≤ 0.04, Fig. 2).

Multivariate Cox regression analysis, with age at diagnosis included as a covariate, confirmed that AJCC stage at primary tumor diagnosis independently predicted mortality following the detection of metastases (Table 4).

**Table 4:** Multivariate Cox regression analysis of hazard ratios for all-cause mortality in metastatic uveal melanoma.

Variable	ß	S.E.ß	Wald Test Statistic	*P* ^†^	exp(ß)	95% CI of exp(ß)
Age^a^	0.01	0.01	4.8	0.03	1.01	Lower: 1.00, Upper: 1.02
AJCC stage^b^	0.16	0.04	12.7	< 0.001	1.17	Lower: 1.07, Upper: 1.27

AJCC, American Joint Committee on Cancer. ß, beta coefficient computed by the Cox proportional hazards analysis. CI, Confidence interval. exp(ß), Exponentiated beta coefficient, representing the hazard ratio. S.E.ß, standard error of the beta coefficient. ^a^ Per increasing year at the time of primary tumor diagnosis.^b^ At the time of primary tumor diagnosis, per increasing step from stage I to IIA, from stage IIA to IIB, from IIB to IIIA, etc. ^†^Holm-Bonferroni-corrected value.

Furthermore, we constructed a Cox model spanning the entire period from primary tumor diagnosis to death or last follow-up, incorporating the number of hepatic metastases detected after the metastasis-free interval as a time-varying covariate. The analysis revealed that the number of hepatic metastases was significantly associated with an increased hazard of death (hazard ratio (HR) 9.41, 95% CI, 6.34–13.97, *P* < 0.001). In contrast, the prognostic significance of AJCC stage at primary tumor diagnosis was not retained (HR 1.05, 95% CI, 0.87–1.27, *P* = 0.61, Table 5), suggesting that the impact of primary tumor size on survival after metastatic detection is related to the number of metastatic lesions.

**Table 5:** Multivariate Cox regression analyses of hazard ratios for all-cause mortality in metastatic uveal melanoma, with the number of metastatic lesions at the time of detection of metastatic disease included as a time-varying covariate.

Variable	ß	S.E.ß	z	*P* ^†^	exp(ß)	95% CI of exp(ß)
Age^a^	0.01	0.01	1.12	0.52	1.01	Lower: 0.99, Upper: 1.03
AJCC stage^b^	0.05	0.10	0.51	0.61	1.05	Lower: 0.87, Upper: 1.27
Number of hepatic metastases^c^	2.24	0.20	11.13	< 0.001	9.41	Lower: 6.34, Upper: 13.97
Number of hepatic metastases × log(*t*_stop_)^d^	-0.83	0.12	-6.72	< 0.001	0.44	Lower: 0.34, Upper 0.56

AJCC, American Joint Committee on Cancer. ß, beta coefficient computed by the Cox Proportional Hazards analysis. CI, Confidence interval. exp(ß), Exponentiated beta coefficient, representing the hazard ratio. S.E.ß, standard error of the beta coefficient. z, Z-statistic, which is the test statistic for the null hypothesis that the corresponding coefficient is zero in the Cox proportional hazards model. ^a^ Per increasing year at the time of primary tumor diagnosis.^b^ At the time of primary tumor diagnosis, per increasing step from stage I to IIA, from stage IIA to IIB, from IIB to IIIA, etc. ^c^ At the time of radiological detection of metastatic disease, per increasing step from a solitary metastasis to two to five, from two to five to six to ten, and from six to ten to > 10 (time-varying covariate). ^d^ *t*_stop_ denotes the end time of each patient’s observation interval (in the counting process format), and log(*t*_stop_) is used to capture the time-dependent change in the effect of the number of metastases. This interaction term allows the hazard ratio for metastatic lesions to vary with follow-up duration. ^†^Holm-Bonferroni-corrected value.

Lastly, a Markov multi-state model was used to evaluate transition dynamics from the initial primary tumor diagnosis to metastasis detection and eventually death. As anticipated, AJCC stage at the time of primary tumor diagnosis significantly influenced the transition from non‐metastatic to metastatic status: each one‐stage increase corresponded to a 37% higher hazard of developing metastases (HR 1.37, 95% CI, 1.09–1.72, *P* = 0.006). For the transition from metastatic disease diagnosis to death, however, AJCC stage lost its significance when the number of hepatic metastases was included as a covariate (Table 6).

**Table 6:** Markov multi-state model hazard ratios for transition dynamics in uveal melanoma.

Transition	Covariate	HR	95% CI of HR	*P* ^†^	Concordance^‡^
Non-metastatic to metastatic disease	AJCC stage^a^	1.37	Lower: 1.09, Upper: 1.72	0.006	0.55
Metastatic disease to death	AJCC stage^a^	1.10	Lower: 0.90, Upper: 1.33	0.36	0.65
	Number of hepatic metastases	1.51	Lower: 1.25, Upper: 1.82	< 0.001	

AJCC, American Joint Committee on Cancer. CI, Confidence Interval. HR, Hazard ratio. ^a^ At the time of primary tumor diagnosis, per increasing step from stage I to IIA, from stage IIA to IIB, from IIB to IIIA, etc.^b^ At the time of radiological detection of metastatic disease, per increasing step from a solitary metastasis to two to five, from two to five to six to ten, and from six to ten to > 10. ^†^Holm-Bonferroni-corrected value. ^‡^C-index, a measure of model performance; higher values indicate better predictive accuracy.

## Discussion

In this study, we found that patients treated for larger primary tumors presented with a greater number of hepatic metastases, even though they did not have more frequent radiologically visible lesions at baseline examinations and underwent radiological surveillance at the same intervals as patients with smaller primary tumors. Furthermore, once metastases were detected, patients with larger primary tumors died sooner than those with smaller tumors. These findings bridge the gap between earlier studies linking larger primary tumor size with shorter time to metastasis on one hand, and between shorter time to metastasis and shorter post-metastasis survival on the other^12,14–16^.

One plausible explanation for the increased number of metastases from larger primary tumors could be that these metastases grow more rapidly, potentially explaining their association with quicker demise. However, caution is warranted, as our findings differ from some previous studies. In 2000, Eskelin and colleagues observed no linear relationship between primary tumor volume and the estimated doubling time of metastases in 37 patients^17^ This underscores the need for further investigations in external, ideally larger, cohorts.

If confirmed, these observations are highly relevant for understanding how primary tumor treatment affects survival. Tumor cells are believed to disseminate early, as supported by the detection of circulating tumor cells and micrometastases in multiple organs of patients who died from unrelated causes^18–20^ Meanwhile, interventions such as enucleation or plaque brachytherapy prevent tumors from reaching higher AJCC stages than they would have in the absence of treatment. It is well-established both that lower stage tumors are associated with decreased mortality, and that the proportion of tumors with aggressive traits such as monosomy 3 and gain of 8q increases with increasing tumor size^4,15,21^ A reasonable interpretation is that a patient’s metastatic risk is largely determined when the tumor is still very small; nevertheless, by diagnosing and treating the tumor promptly, we may avert the additional risk conferred by continued tumor growth.

We acknowledge the validity of theories suggesting that a patient’s lifespan from diagnosis to death from metastases could be determined by micrometastases and genetic traits established at an early stage^19,22,23^ The longer interval between primary tumor diagnosis and metastatic death for smaller tumors might occur because these tumors are detected earlier in their progression. In contrast, larger tumors may have been growing undetected for a longer period, which could explain the shorter time from diagnosis to death for patients with larger tumors, as well as the observation of a greater number of metastases upon the initial detection of metastatic disease^24^ However, this reasoning, which assumes that all metastases grow at similar rates irrespective of primary tumor size and that any differences in survival time are merely a result of lead time bias, does not fully explain why patients with metastatic disease experience shorter survival when their primary tumor was diagnosed at a more advanced stage. Indeed, a recent meta-analysis indicates that tumor doubling time decreases as primary tumor grow larger^25^.

An alternative explanation is therefore that metastases originating from larger, more advanced primary tumors tend to grow more rapidly and are more immediately life-threatening than metastases from smaller, less advanced primary tumors. Thus, our findings suggest that a potential survival benefit of primary tumor treatment is to prevent the tumor from advancing to a higher AJCC stage and acquiring more malignant genetic traits, thereby reducing the aggressiveness and growth rate of the metastases it seeds.

These findings also underscore the potential survival benefits of timely primary tumor treatment in uveal melanoma^26,27^ While it is evident that treatment cannot be initiated for tumors that have not yet been detected, and that some observation for growth is necessary to differentiate between benign and malignant choroidal melanocytic lesions, once a diagnosis of melanoma is established, delaying treatment may be detrimental. Treatment should be administered as soon as practically possible after diagnosis to minimize the risk of tumor progression to a more advanced AJCC stage. It is also important to note that small tumors that grow slowly will naturally take longer to progress to a higher AJCC stage, implying that delaying treatment for these lesions may have minimal or no impact on prognosis compared to rapidly growing tumors^25,28^ Conversely, while larger tumors are generally associated with more aggressive behavior, some small tumors may possess aggressive genetic mutations, and some larger tumors may have characteristics associated with a more favorable prognosis^15^ This variability highlights the need for a nuanced approach to treatment decisions, considering both tumor size and its molecular behavior. Many institutions tailor their radiological surveillance programs based on perceived metastatic risk, determined by factors such as AJCC stage, chromosome 3 status, gene expression profiling, or other prognostic markers^29^ While it may seem logical to recommend more frequent radiological examinations for patients with markers of aggressive disease, and less frequent or no examinations for those with markers of lower metastatic risk (e.g., AJCC stage I, disomy 3, or *EIF1AX* mutation), it is important to note that, to date, there is no evidence that surveillance improves survival in uveal melanoma. Our observations support this, as there was no significant difference in the number of metastatic lesions at the presentation of metastatic disease between patients who did and did not undergo radiological surveillance.

The next few years may be pivotal in this regard, as new treatments for metastatic disease could potentially improve survival rates, thereby increasing the value of early detection of metastatic lesions.

## Limitations

This study has several limitations beyond those already discussed. Firstly, the results are derived from retrospective observational cohorts, which inherently limits the ability to draw definitive conclusions about causality. The potential survival benefit of early treatment versus observation of small primary uveal melanomas has been debated within the field^30^ Most previous studies have not found a significant association between the stage or size of the primary tumor at initial diagnosis and survival in patients with metastatic disease, suggesting caution in interpreting such associations^7,8,12,13^ To truly determine whether primary tumor treatment confers a survival benefit in uveal melanoma, a large randomized clinical trial would be necessary, comparing outcomes between a treated group and an untreated group. However, such a trial would raise serious ethical concerns and is unlikely to ever be conducted. Additionally, the validity of our findings relies on the accuracy of the underlying data, which was obtained from medical charts, treatment records, and cause of death registries. Although efforts were made to cross-verify information from cause of death registries with medical records, the possibility of misclassification remains. Such errors could have influenced the survival outcomes reported in this study.

Thirdly, the assessment of the dimensions and anatomical extent of primary tumors and metastases varies by measurement method. For primary tumors, methods include fundus photography, ultrasonography, and gross pathological examination, while for metastases, ultrasound, MRI, CT, and PET/CT are used. Gross pathological examination, commonly performed on enucleated eyes, involves various techniques that may affect measurement consistency. Some pathologists measure the chord length of the tumor’s transillumination shadow with a caliper, while others use a flexible ruler to measure arc dimensions, potentially yielding different results, especially in larger tumors (> 10 mm). Measurements can also vary after the globe is opened, depending on sectioning and whether taken fresh or after formalin fixation, which causes tissue shrinkage—affecting thickness more than basal diameter. Due to these discrepancies, it is uncertain how well pathological measurements reflect the true tumor dimensions compared to clinical measurements obtained with current methods.

Fourth, patient age was included as a covariate in regression analyses, despite ongoing debate about its association with prognosis, especially when considering competing risks and other prognostic factors in multivariate analyses^2,31^ However, age-related factors, such as comorbidities and overall health status, may affect both disease progression and a patient’s ability to tolerate aggressive treatments for metastases. Additionally, age has been linked to aggressive genetic traits, such as *BAP1* mutation and aggressive gene expression profiles^32^ Older patients may present with more advanced disease within the same stage category due to a longer potential duration of tumor growth, supporting our decision to include age as a covariate.

Fifth, we lacked data on the genetic or cytogenetic characteristics of both primary tumors and metastases. Additionally, data on the number of metastases were available for only 144 patients, and the size of these lesions was not documented. Information regarding the involvement of organs other than the liver at the time of first metastatic detection was also unavailable. The size and distribution of metastatic lesions are important prognostic factors in metastatic disease, and including these data in regression analyses might have altered the results. However, the number of hepatic metastases, which is associated with the presence of miliary metastases, also has substantial prognostic value^7,24,33^ Thus, we believe that the absence of data on the size of the largest metastatic lesion, and the potential involvement of other organs, likely had a limited impact on our findings.

Finally, the examined cohorts in our study were heterogeneous, with a median overall survival of 0.6 years in the Rotterdam cohort and 1.3 years in the Stockholm cohort. Nevertheless, it could be argued that the consistent association between larger primary tumor size and survival in metastatic disease—even under varying epidemiological conditions—supports the robustness of our findings.

## Conclusions

This study found that patients treated for larger primary tumors presented with a greater number of hepatic metastases and, once metastases were detected, had shorter survival than those with smaller tumors. These findings suggest that treating the primary tumor, thereby preventing its progression to a more advanced AJCC stage, may offer a survival benefit for some patients, by potentially reducing the aggressiveness and growth rate of subsequent metastases.

## Methods

### Aim of the study

The aim of this study was to examine the prognostic implication of primary tumor size for survival in metastatic disease.

### Patients and study design

We collected data on all patients who developed metastatic disease after being diagnosed with primary uveal melanoma at either the Erasmus University Medical Center and the Rotterdam Eye Hospital, Rotterdam, The Netherlands, between 1993 and 2021, or the Ocular Oncology Service, St. Erik Eye Hospital, Stockholm, Sweden, between 1980 and 2021. A total of 626 patients met the following inclusion criteria:

Inclusion criteria:

1. Data available in treatment registries at the respective institution.
2. Clinically or histopathologically confirmed diagnosis of choroidal and/or ciliary body melanoma at the time of primary tumor diagnosis.
3. Enrollment in a surveillance program with periodic liver examinations for a minimum of 5 years after primary tumor diagnosis (using contrast-enhanced ultrasound, computed tomography [CT], or magnetic resonance imaging [MRI]).
4. Radiologically detected metastases in the liver and/or other organs.
5. Availability of CT or MRI images.

Exclusion criteria:

1. Iris melanoma (*n* = 0).
2. Lack of recorded primary tumor thickness or LBD at the time of primary tumor diagnosis (*n* = 29).
3. Unknown anatomical extent, specifically CBI or EXE, at the time of primary tumor diagnosis (*n* = 0).
4. Unrecorded location of metastases (*n* = 51).
5. Uncertain exact date of radiological detection of metastases, including cases where dates were not specified in referrals, medical notes, or radiological image files, or where the diagnosis was indeterminable due to unclear findings in one exam followed by an established diagnosis in a subsequent exam (*n* = 213).

After applying these criteria, 333 patients remained in the final cohort, of which 128 were from Rotterdam and 205 from Stockholm. The date of the first radiological detection of metastases was used as the date of metastasis. Data on the number of patients who underwent biopsy for histopathological confirmation of metastases were not available. Of the 128 patients from Rotterdam, 123 were included in a previous study, with metastases to the liver being observed in 96% of cases^24^ For the 123 patients in the Rotterdam sample, and for 21 of the patients in the Stockholm samle, data were available on the number of metastases present at the initial CT or MRI scan when the first metastases were observed.

The study was approved by the Swedish Ethical Review Authority (reference 2023-07537-02) and adhered to the tenets of the Declaration of Helsinki. Informed consent was waived by the Swedish Ethical Review Authority because the study relied on retrospective, pre-collected data. No sensitive information was shared between the institutions, and no new collection of identifiable information was conducted, including patients’ names, identification numbers, addresses, contact details, or photographs. No interventions, testing, or examinations were performed, and no analyses of biological tissues were conducted.

### Statistical analyses

Statistical significance was defined as *P* < 0.05, and all *P* values were two-sided. Holm-Bonferroni corrections were applied to all reported *P* values. Kaplan-Meier survival curves were generated, and multivariate and time-varying Cox regression analyses were performed using the survival and survminer packages in R (version 4.4.1, The R Foundation for Statistical Computing, Vienna, Austria). Survival distributions across AJCC stages at primary tumor diagnosis were compared using the log-rank test for trend. Additionally, patients were categorized based on whether the largest basal diameter of the primary tumor was larger or smaller than 16 mm. This threshold aligns with the definition of large tumors in the Collaborative Ocular Melanoma Study (COMS) and represents a common cutoff where enucleation is often favored over eye-preserving treatments such as plaque brachytherapy^34^ To examine the transition dynamics from primary tumor diagnosis to metastasis detection and subsequent death, a Markov multi-state model was constructed using the mstate and survival packages in R. The proportional hazards assumption was assessed by inspecting log-minus-log survival curves; the assumption was considered satisfied if the curves were parallel and did not cross. Tumor volume was estimated using a formula consistent with previously described methods, where LBD represents the largest basal tumor diameter:^35,36^

$$\:\text{E}\text{s}\text{t}\text{i}\text{m}\text{a}\text{t}\text{e}\text{d}\:\text{v}\text{o}\text{l}\text{u}\text{m}\text{e}\:\text{o}\text{f}\:\text{t}\text{u}\text{m}\text{o}\text{r}=\frac{{\uppi\:}}{6}\times\:t\times\:\text{L}\text{B}\text{D}\times\:(\text{L}\text{B}\text{D}\times\:0.85)$$

## Electronic supplementary material

Below is the link to the electronic supplementary material.

Supplementary Material 1

## Data Availability

Data, including the Rotterdam and Stockholm samples, are available upon reasonable request from the corresponding author, subject to approval from the Swedish Ethical Review Authority.
