# Supplementary material for: Higher AJCC stage at primary tumor diagnosis may predict shorter survival in metastatic uveal melanoma
Source: Sci Rep. 2025 Jun 4;15:19557. doi: 10.1038/s41598-025-03961-1 (PMC12137540; doi:10.1038/s41598-025-03961-1)
Supplement: Supplementary file 1 — Supplementary Material 1 [file 41598_2025_3961_MOESM1_ESM.pdf]

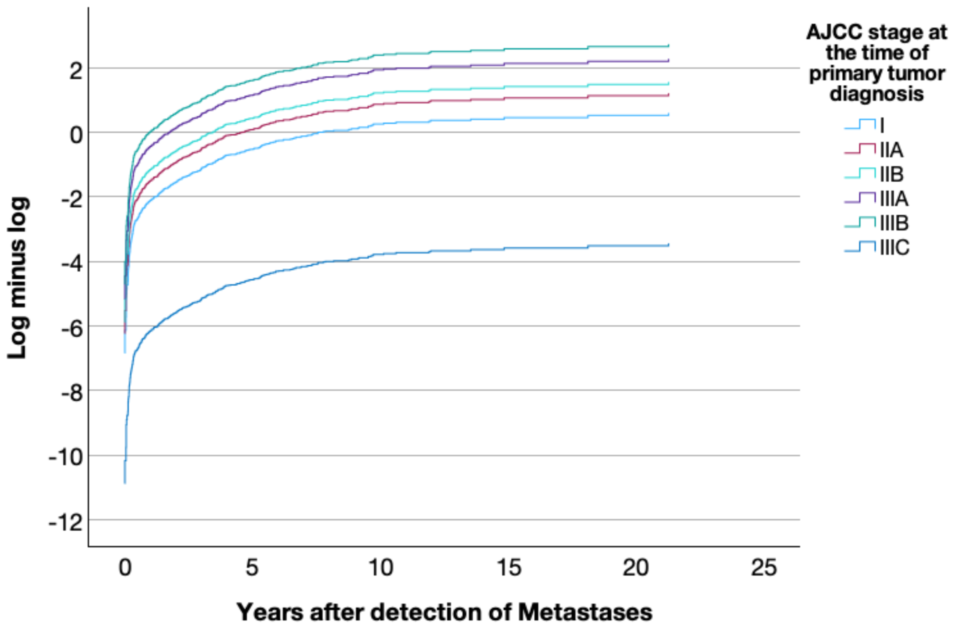

Supplementary Figure S1. Log-Minus-Log Survival Curve
This plots illustrates the log-minus-log survival curves for the AJCC stage at the time of primary tumor diagnosis. The parallel nature of the curves without any crossings indicates that the proportional hazards assumption is reasonably satisfied for the AJCC stage covariate. However, the log-minus-log survival curve for patients initially diagnosed at stage IIIC shows a noticeably larger gap to stage IIIB compared to the gaps between other adjacent stages. This observation suggests a greater difference in baseline hazard or survival experience between stage IIIC and stage IIIB patients. Nevertheless, this difference does not constitute a violation of the proportional hazards assumption, as it reflects a consistent proportional difference over time.
